# Dopamine suppresses persistent network activity via D_1_-like dopamine receptors in rat medial entorhinal cortex

**DOI:** 10.1111/ejn.12125

**Published:** 2013-01-22

**Authors:** Elizabeth W Mayne, Michael T Craig, Chris J McBain, Ole Paulsen

**Affiliations:** 1 Department of Physiology, Anatomy and Genetics, University of OxfordOxford, UK; 2 Program in Developmental Neurobiology, Eunice Kennedy Shriver National Institute of Child Health and Human Development, National Institutes of HealthBethesda, MD, USA; 3 Department of Physiology, Development and Neuroscience, Physiological Laboratory, University of CambridgeCambridge, UK

**Keywords:** cortex, slow oscillation, UP states

## Abstract

Cortical networks display persistent activity in the form of periods of sustained synchronous depolarizations (‘UP states’) punctuated by periods of relative hyperpolarization (‘DOWN states’), which together form the slow oscillation. UP states are known to be synaptically generated and are sustained by a dynamic balance of excitation and inhibition, with fast ionotropic glutamatergic excitatory and GABAergic inhibitory conductances increasing during the UP state. Previously, work from our group demonstrated that slow metabotropic GABA receptors also play an important role in terminating the UP state, but the effects of other neuromodulators on this network phenomenon have received little attention. Given that persistent activity is a neural correlate of working memory and that signalling through dopamine receptors has been shown to be critical for working memory tasks, we examined whether dopaminergic neurotransmission affected the slow oscillation. Here, using an *in vitro* model of the slow oscillation in rat medial entorhinal cortex, we showed that dopamine strongly and reversibly suppressed cortical UP states. We showed that this effect was mediated through D_1_-like and not D_2_-like dopamine receptors, and we found no evidence that tonic dopaminergic transmission affected UP states in our model.

## Introduction

Neuronal networks display persistent activity, an important form of circuit dynamics thought to underlie phenomena such as working memory (reviewed by Major & Tank, 2004). One example of persistent activity is seen during the slow oscillation, where neurons oscillate between periods of sustained depolarization and firing (‘UP states’) punctuated by epochs of hyperpolarization and reduced firing (‘DOWN states’). The slow oscillation can be observed *in vivo* in anaesthetized (Steriade *et al*., 1993) or naturally sleeping (Contreras *et al*., 1996) states as well as during quiet wakefulness (Petersen, 2003). UP and DOWN states (UDS) can also be observed using reduced *in vitro* preparations such as slices containing the visual cortex (Sanchez-Vives & McCormick, 2000) or entorhinal cortex (Cunningham *et al*., 2006; Mann *et al*., 2009). Insights from these *in vitro* studies show that UP states are associated with an increase in both excitatory and that inhibitory neurotransmission (Sanchez-Vives & McCormick, 2000), and that inhibitory conductances dynamically scale to balance excitatory conductances, both *in vitro* (Shu *et al*., 2003) and *in vivo* (Haider *et al*., 2006).

Most studies of the circuit dynamics involved in generating UDS have focussed on fast, ionotropic neurotransmission and less on the role played by slower metabotropic receptors. We previously reported that fast GABA_A_ receptor-mediated inhibition balances the UP state, whereas slow GABA_B_ receptor-mediated inhibition is important for the termination of the UP state (Mann *et al*., 2009), but whether other neuromodulators affect the slow oscillation has yet to be determined. Working memory and its neural correlate in the prefrontal cortex, persistent activity, are known to be dependent upon dopamine (Brozoski *et al*., 1979). In the prefrontal cortex, dopamine can reduce extracellular GABA concentrations (Grobin & Deutch, 1998) via a mechanism involving both D_1_-like and D_2_-like dopamine receptors (Bouthenet *et al*., 1987; Gao *et al*., 2001). Much like the prefrontal cortex, the medial entorhinal cortex (mEC) receives multimodal input from many brain regions. The mEC forms one of the main input and output systems to the hippocampus (Canto *et al*., 2008), so persistent activity in the mEC may be involved in cognitive processes such as working memory and spatial navigation. Diffuse neuromodulatory systems, such as dopaminergic projections, might therefore also be expected to modulate the persistent activity of UDS in the mEC.

Using a model of UDS in submerged mEC slices (Mann *et al*., 2009), we sought to determine whether the dopaminergic system affected the slow oscillation. We found that dopamine rapidly and reversibly suppressed the incidence of UP states in rat mEC, and that this effect was mediated through D_1_-like dopamine receptors. We found no evidence for the involvement of D_2_-like receptors in the dopamine-mediated suppression of persistent activity.

## Materials and methods

### Animals

All experiments were conducted in accordance with the UK Animals Scientific Procedures Act (1986) and were subject to local ethical review by the University of Oxford and the University of Cambridge. Wistar rats (Harlan, UK) were used in all experiments.

### Slice preparation and electrophysiology

Horizontal slices (400 μm) containing the mEC were prepared from postnatal day 14–21 rats of both sexes after decapitation under deep isoflurane-induced anaesthesia. Slices were cut in ice-cold (< 4 °C) standard artificial cerebrospinal fluid containing (in mm): 126 NaCl, 3–3.5 KCl, 1.25 NaH_2_PO_4_, 2 MgSO_4_, 2 CaCl_2_ and 26 NaHCO_3_, and were incubated at room temperature (22 − 26 °C) for 1 h in interface conditions with standard artificial cerebrospinal fluid, before being transferred to modified artificial cerebrospinal fluid with reduced MgSO_4_ (1 mm) and CaCl_2_ (1.2 mm). Slices were maintained in interface conditions prior to recording; they were then mounted on a coverslip (coated with 0.1% poly-l-lysine in ultrapure H_2_O) and transferred to a submerged-style recording chamber where they were superfused with modified artificial cerebrospinal fluid at 4–5 mL/min at 32–34 °C. The maintenance of slices in interface conditions was critical for the development of spontaneous UDS, and the use of poly-l-lysine coverslips allowed slices to be mechanically stable under high perfusion rates and also improved laminar flow across the slice; these conditions are known to promote spontaneous network activity in submerged slices (Hajos *et al*., 2009).

Whole-cell current-clamp recordings were made from principal cells in layer 3 of the mEC, using glass pipettes pulled from standard borosilicate glass (6–8 MΩ) containing (in mm): 110 K gluconate, 40 HEPES, 2 ATP-Mg, 0.3 GTP, 4 NaCl and 2–4 mg/mL biocytin (pH 7.2–7.3, osmolarity 275–290 mosmol/L). Membrane potential values were not corrected for the liquid junction potential.

### Data acquisition and analysis

Data were recorded using an Axon Multiclamp 700B amplifier (Molecular Devices) and low-pass filtered at 2 kHz. The signal was digitized at 5 kHz using an Instrutech ITC-18 on a PC running Igor Pro (Wavemetrics Inc., Lake Oswego, OR, USA) using procedures written in-house. UDS transitions were monitored automatically using an algorithm that detected changes in DC membrane potential and membrane potential fluctuations using a moving average window method (Craig, 2011). All detected UP states were confirmed by visual inspection. Statistical comparisons were made using anova with *post hoc* Bonferroni multiple-comparison correction, or Student's two-sample and paired *t*-tests as appropriate. Unless otherwise stated, all values are given as mean ± SEM. All data and statistical analyses were carried out using either Igor Pro or GraphPad Prism (GraphPad, San Diego, CA, USA).

### Drugs and chemicals

Raclopride and SCH23390 were purchased from Tocris Bioscience. All other chemicals were purchased from Sigma-Aldrich.

## Results

### UP states occur spontaneously in the medial entorhinal cortex and are suppressed by bath application of dopamine

**FIG. 1:**
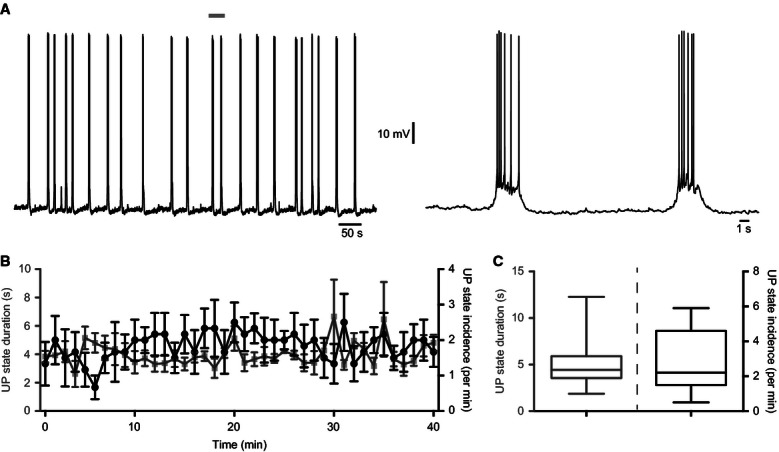
UP states in the mEC are stable over time. (A) Whole-cell current-clamp recording from a layer 3 principal cell showing that UDS are stable over long periods. Area marked by grey bar is shown on the right on an expanded time scale. (B) The duration (grey squares) and incidence (black circles) of UP states did not change over time (*n* = 6). (C) Distribution of mean UP state duration (left) and incidence (right). The mean UP state duration was 5.2 ± 0.4 s and the mean UP state incidence was 2.9 ± 0.3/min (*n* = 37 slices). Whiskers on the box plots represent the maximum and minimum values.

**FIG. 2:**
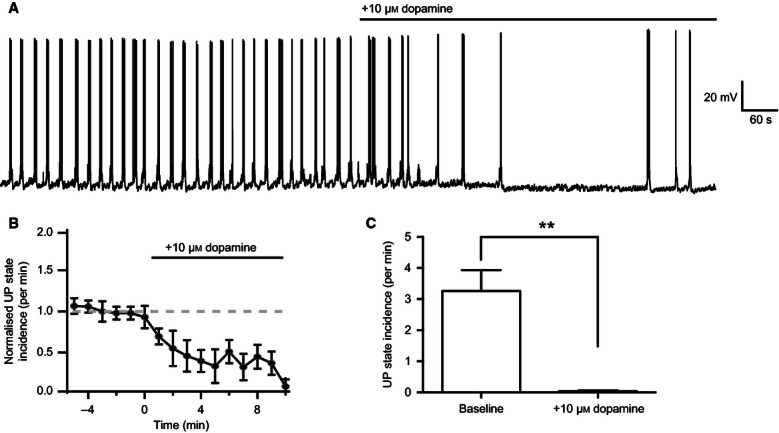
Dopamine suppresses the incidence of UP states. (A) Example recording showing the reduction in the incidence of UP states after bath application of 10 μm dopamine. (B) Time course of the depression of UP states over 10 min after start of wash-in (*n* = 14). (C) After 10 min, dopamine had significantly reduced the frequency of UP states (*n* = 5). **P < 0.01, paired *t*-test.

Whole-cell current-clamp recordings made from principal cells in layer 3 of the mEC were used to monitor UDS. UP states occurred spontaneously with an incidence of 2.9 ± 0.3/min and a mean duration of 5.2 ± 0.4 s (*n* = 37 slices, Fig. 1A and C). The mean spiking frequency of principal cells during UP states was 0.6 ± 0.2 Hz. The incidence of UP states was stable for at least 40 min after whole-cell configuration was achieved (Fig. 1B). Bath application of 10 μm dopamine reduced the incidence of UP states (Fig. 2A and B); after 10 min, spontaneous UP states were almost entirely suppressed (UP state incidence, baseline vs. 10 μm dopamine: 3.3 ± 0.67/min vs. 0.04 ± 0.03/min; *t*_4_ = 4.8, *P* = 0.0089; *n* = 5; paired *t*-test; Fig. 2C). Bath application of dopamine caused a small but significant hyperpolarization of the resting membrane potential (baseline vs. 10 μm dopamine: −62.1 ± 0.16 vs. −64.3 ± 0.19 mV; *t*_4_ = 2.9, *P* = 0.044; *n* = 5; two-tailed paired *t*-test). We next asked which type of dopamine receptor, D_1_-like or D_2_-like, mediates the effects of dopamine on UDS.

### Dopaminergic suppression of UP states is mediated by D_1_-like dopamine receptors

**FIG. 3:**
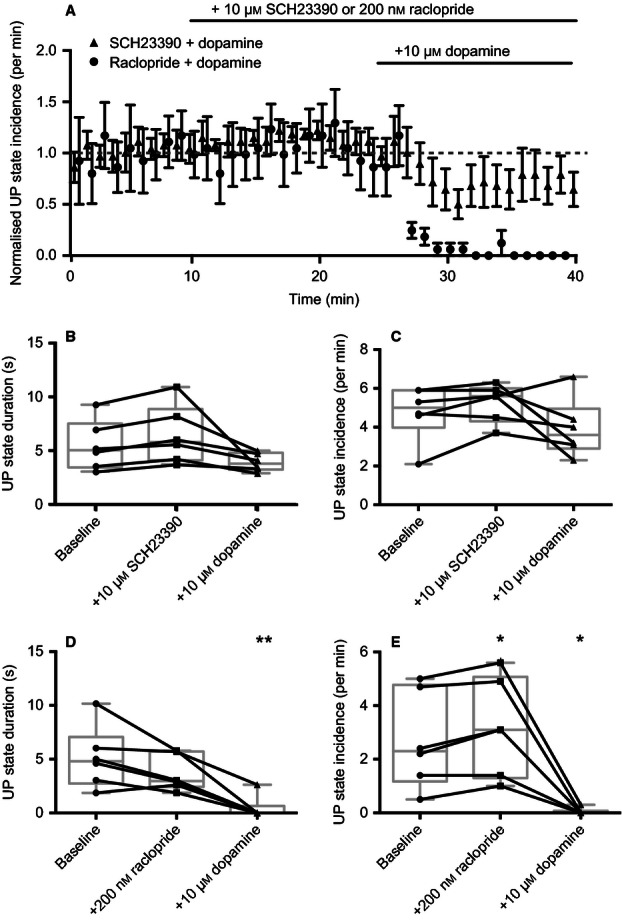
Dopamine-induced suppression of persistent activity is mediated via D_1_-like receptors. (A) The D_1_-like receptor antagonist SCH23390 prevented dopamine-induced suppression of UP states, whereas the D_2_-like receptor antagonist raclopride did not. (B) In the presence of SCH23390, no significant difference was observed in UP state duration upon application of dopamine. (C) In the presence of SCH23390, no significant difference was observed in UP state incidence upon application of dopamine. (D) In the presence of raclopride, dopamine significantly reduced the duration of UP states. (E) In the presence of 200 nm raclopride, dopamine also significantly reduced the incidence of UP states (*n* = 6). *P < 0.05, **P < 0.01; repeated-measures anova.

To investigate the dopamine receptor subtype involved in suppressing persistent activity, we repeated the previous experiment in the presence of either the D_1_-like receptor antagonist SCH23390 or the D_2_-like receptor antagonist raclopride. Bath application of 10 μm SCH23390 prior to bath application of dopamine prevented the suppression of UP states (Fig. 3A), with no significant effect on either the duration (baseline vs. +10 μm SCH23390 vs. +10 μm dopamine: 5.5 ± 0.94 vs. 6.4 ± 1.10 vs. 3.9 ± 0.33 s; *F*_2,5_ = 4.9, *P* = 0.077; *n* = 6; repeated-measures anova; Fig. 3B) or incidence (baseline vs. +10 μm SCH23390 vs. +10 μm dopamine: 4.8 ± 0.58/min vs. 5.3 ± 0.40/min vs. 3.9 ± 0.61/min; *F*_2,5_ = 2.3, *P* = 0.19; *n* = 6; repeated-measures anova; Fig. 3C) of UP states. Conversely, bath application of 200 nM raclopride failed to prevent the subsequent suppression of UP states after the application of dopamine (Fig. 3A), which significantly reduced UP state duration (baseline vs. +200 nm raclopride vs. +10 μm dopamine: 5.1 ± 1.17 vs. 3.7 ± 0.68 vs. 0.4 ± 0.44 s; *F*_2,5_ = 15.5, *P* = 0.0091; *n* = 6; multiple comparisons after repeated-measures anova; Fig. 3D) and incidence (baseline vs. +200 nm raclopride vs. +10 μm dopamine: 2.7 ± 0.73/min vs. 3.2 ± 0.74/min vs. 0.05 ± 0.05/min; *F*_2,5_ = 16.6, *P* = 0.0082; *n* = 6; multiple comparisons after repeated-measures anova; Fig. 3E). We observed no significant relationship between the baseline UP state incidence and the change in UP state incidence after bath application of dopamine alone (r = −0.50, *P* = 0.39; *n* = 5; Pearson's correlation test), dopamine and raclopride (r = 0.62, *P* = 0.18; *n* = 6) or dopamine and SCH23390 (r = 0.06, *P* = 0.92, *n* = 6; Pearson's correlation test). We conclude that dopamine-induced suppression of persistent activity is mediated through D_1_-like receptors.

### Application of a D_1_-like receptor antagonist can reverse dopamine-induced suppression of persistent activity

**FIG. 4:**
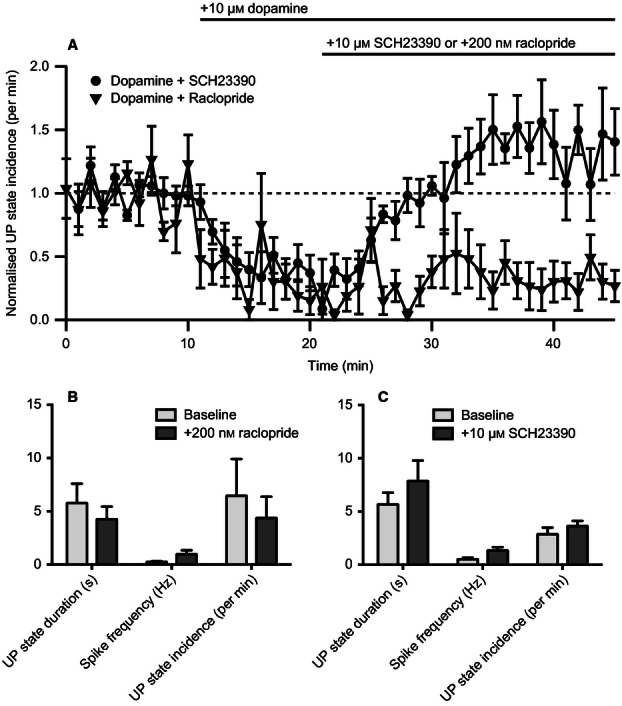
A D_1_-like antagonist can reverse dopamine-induced suppression of persistent activity. (A) Application of SCH23390 could reverse dopamine-induced suppression of UP states, but application of raclopride could not (*n* = 6). (B and C) SCH233390 and raclopride alone had no significant effect on UP state duration, incidence or firing frequency (*n* = 6). Repeated-measures anova.

Given that the D_1_-like receptor antagonist SCH23390 could prevent dopamine from suppressing UP states, we next sought to determine whether this suppression was reversible. After recording spontaneous UDS for 10 min, 10 μm dopamine was bath-applied. Subsequently, we then applied either 10 μm SCH23390 or 200 nm raclopride. Application of SCH23390 significantly reversed the suppression of UP states, whereas raclopride had no effect (normalized incidence of UP states at 25 min after washing antagonist, 10 μm SCG23390 vs. 200 nm raclopride: 1.4 ± 0.26/min vs. 0.30 ± 0.14/min; *t*_10_ = 3.7, *P* = 0.004; *n* = 6; two-tailed Student's *t*-test; Fig. 4A). As well as reversing the dopamine-induced suppression of UP states, it appeared that 10 μm SCH23390 might have increased the incidence above baseline values. However, this increase in UP state incidence was not statistically significant (*t*_5_ = 1.5, *P* = 0.18; one-sample *t*-test; *h*_0_ = normalized UP state incidence after SCH22390 application was 1).

In the previous experiment, application of 200 nm raclopride appeared to cause a small but significant increase in UP state incidence (Fig. 3E). To determine whether there was any tonic dopaminergic activity affecting the UP states, we bath-applied either 10 μm SCH23390 or 200 nm raclopride for 40 min to observe the effect on UDS properties. Raclopride had no significant effect on UP state duration (baseline vs. 200 nm raclopride: 5.8 ± 1.8 vs. 4.3 ± 1.2 s; *t*_5_ = 1.6, *P* = 0.17; *n* = 6; two-tailed paired *t*-test; Fig. 4B), incidence (baseline vs. 200 nm raclopride: 6.5 ± 3.5/min vs. 4.4 ± 2.1/min; *t*_5_ = 1.4, *P* = 0.23; *n* = 6; two-tailed paired *t*-test; Fig. 4B) or spiking frequency (baseline vs. 200 nm raclopride: 0.25 ± 0.07 vs. 0.97 ± 0.37 Hz; *t*_5_ = 1.9, *P* = 0.12; *n* = 6; two-tailed paired *t*-test; Fig. 4B). Similarly, SCH23390 had no significant effect on UP state duration (baseline vs. 10 μm SCH23390: 5.7 ± 1.1 vs. 7.9 ± 1.9 s; *t*_5_ = 2.1, *P* = 0.092; *n* = 6; two-tailed paired *t*-test; Fig. 4C), incidence (baseline vs. 10 μm SCH23390: 2.9 ± 0.62/min vs. 3.6 ± 0.49/min; *t*_5_ = 1.9, *P* = 0.12; *n* = 6; two-tailed paired *t*-test; Fig. 4C) or spiking frequency (baseline vs. 10 μm SCH23390: 0.52 ± 0.15 vs. 1.3 ± 0.32 Hz; *t*_5_ = 2.3, *P* = 0.070; *n* = 6; two-tailed paired *t*-test; Fig. 4C).

These data suggest that there was no detectable tonic effect of dopamine in our preparation, and we conclude that phasic application of dopamine can powerfully but reversibly block persistent activity in the mEC through a mechanism mediated by D_1_-like dopamine receptors.

## Discussion

The slow oscillation is a synaptically-driven oscillation, where UP states are generated by recurrent activity mediated via a dynamic balance of excitatory and inhibitory conductances (Sanchez-Vives & McCormick, 2000; Shu *et al*., 2003; Cunningham *et al*., 2006; Haider *et al*., 2006). Whereas the contributions of fast, ionotropic excitatory (Sanchez-Vives & McCormick, 2000; Compte *et al*., 2003; Shu *et al*., 2003) and inhibitory (Hasenstaub *et al*., 2005; Haider *et al*., 2006) conductances to generating neocortical UP states are well understood, the effects that neuromodulators have in modulating persistent activity are less so. Previously, work from our group showed that slow GABA_B_ receptors are involved in both the spontaneous and evoked termination of UP states (Mann *et al*., 2009), and here we have shown that dopamine can strongly suppress UP states.

We found that blockade of D_1_-like receptors with the antagonist SCH23390 prevented the dopamine-induced suppression of UP states, although there still appeared to be a small, but non-significant reduction in UP state incidence (Fig. 3A). SCH23390 is also an agonist at 5-HT_1C_ and 5-HT_2C_ receptors so it is possible that activation of these receptors may have had an effect on network oscillations. However, we saw no evidence for significant modulation of UP states by application of dopaminergic antagonists alone, suggesting that the off-target activation of 5HT_1C_ and 5HT_2C_ receptors had a minimal effect in our model.

Although it has been shown that, in the striatal spiny neurons, activation of D_2_ receptors can truncate UP states (Plotkin *et al*., 2011), we found that the effects of dopamine on mEC UP states were mediated via D_1_-like receptors. As dopamine is necessary for working memory in the prefrontal cortex and can lower extracellular GABA concentrations (Brozoski *et al*., 1979; Grobin & Deutch, 1998), one might have expected dopamine to increase excitation and thus enhance persistent activity, yet we saw the opposite result. However, even within the prefrontal cortex, the effects of dopamine can appear contradictory and dependent upon experimental conditions (Seamans & Yang, 2004). We chose a concentration of dopamine (10 μm) that was at the lower end of those reported in the literature, but that should still have activated both D_1_-like and D_2_-like receptors. There are a number of potential mechanisms that could explain why dopamine suppresses persistent activity. First, an early study in the entorhinal cortex found that dopamine could inhibit layer 5 pyramidal cells by acting via D_1_-like receptors to increase *I*_H_ (Rosenkranz & Johnston, 2006), and a similar effect could potentially act on layer 3 principal cells in our study. D_1_-like receptors also depolarize neocortical fast-spiking interneurons (Towers & Hestrin, 2008); a shift in the excitatory/inhibitory balance caused by dopamine could perturb network activity. Other studies report that activation of D_1_-like receptors can actually reduce evoked inhibitory postsynaptic currents in the prefrontal cortex (Gonzalez-Islas & Hablitz, 2001). D_1_/D_5_ receptors have also been shown to increase the *N*-methyl-d-aspartate component of excitatory postsynaptic currents in prefrontal cortex pyramidal cells (Seamans *et al*., 2001), which could also affect persistent activity by altering the excitatory/inhibitory balance of the network. Dopamine's effects on network activity are likely to vary substantially from one region of the brain to another, depending on both the cellular and laminar distribution of D_1_-like vs. D_2_-like receptors. An additional degree of variability may arise due to differential expression of the intracellular targets of activated dopamine receptors between different areas of the cortex.

A more recent study reported that, in prefrontal cortex slices, bath application of dopamine selectively increased inhibitory over excitatory currents and, using calcium imaging, dopamine was observed to inhibit the spread of local activity via D_1_-like dopamine receptor activation (Bandyopadhyay & Hablitz, 2007). Dopamine also inhibits carbachol-induced gamma oscillations in rat hippocampal slices, through a mechanism that also acts via D_1_-like dopamine receptors (Weiss *et al*., 2003). Taken together with the results from our study, it would seem that dopamine can strongly inhibit network activity by acting through D_1_-like receptors. Further questions remain, e.g. are the actions of dopamine mediated through a specific type of interneuron, or do they come from a general increase in inhibitory tone? These questions have functional implications; understanding the effects of dopamine on the network, as opposed to neuronal level, may help give insights into the progression and treatment of brain disorders.

Given the diversity of neuronal functions that can be attributed to dopamine, which vary depending on the location and time course of release (Schultz, 2007), in addition to the number and diversity of dopamine receptors present throughout the brain, understanding the effects of dopamine on network activity can be challenging. Although we have shown here that dopamine can strongly suppress persistent network activity in the mEC, further work will be needed to determine the mechanism(s) by which this effect is mediated.

## Abbreviations

mEC: medial entorhinal cortex
UDS: UP and DOWN states

## References

[b1] Bandyopadhyay S, Hablitz JJ (2007). Dopaminergic modulation of local network activity in rat prefrontal cortex. J. Neurophysiol.

[b2] Bouthenet ML, Martres MP, Sales N, Schwartz JC (1987). A detailed mapping of dopamine D-2 receptors in rat central nervous system by autoradiography with [^125^I]iodosulpride. Neuroscience.

[b3] Brozoski T, Brown R, Rosvold H, Goldman P (1979). Cognitive deficit caused by regional depletion of dopamine in prefrontal cortex of rhesus monkey. Science.

[b4] Canto CB, Wouterlood FG, Witter MP (2008). What does the anatomical organization of the entorhinal cortex tell us?. Neural Plast.

[b5] Compte A, Sanchez-Vives MV, McCormick DA, Wang XJ (2003). Cellular and network mechanisms of slow oscillatory activity (< 1 Hz) and wave propagations in a cortical network model. J. Neurophysiol.

[b6] Contreras D, Timofeev I, Steriade M (1996). Mechanisms of long-lasting hyperpolarizations underlying slow sleep oscillations in cat corticothalamic networks. J. Physiol.

[b7] Craig MT (2011). The cortical slow oscillation: the role of slow GABAergic inhibition in mediating the UP-to-DOWN state transition.

[b8] Cunningham MO, Pervouchine DD, Racca C, Kopell NJ, Davies CH, Jones RS, Traub RD, Whittington MA (2006). Neuronal metabolism governs cortical network response state. Proc. Natl. Acad. Sci. USA.

[b9] Gao W-J, Krimer LS, Goldman-Rakic PS (2001). Presynaptic regulation of recurrent excitation by D1 receptors in prefrontal circuits. Proc. Natl. Acad. Sci. USA.

[b10] Gonzalez-Islas C, Hablitz JJ (2001). Dopamine inhibition of evoked IPSCs in rat prefrontal cortex. J. Neurophysiol.

[b11] Grobin AC, Deutch AY (1998). Dopaminergic regulation of extracellular gamma-aminobutyric acid levels in the prefrontal cortex of the rat. J. Pharmacol. Exp. Ther.

[b12] Haider B, Duque A, Hasenstaub AR, McCormick DA (2006). Neocortical network activity in vivo is generated through a dynamic balance of excitation and inhibition. J. Neurosci.

[b13] Hajos N, Ellender TJ, Zemankovics R, Mann EO, Exley R, Cragg SJ, Freund TF, Paulsen O (2009). Maintaining network activity in submerged hippocampal slices: importance of oxygen supply. Eur. J. Neurosci.

[b14] Hasenstaub A, Shu Y, Haider B, Kraushaar U, Duque A, McCormick DA (2005). Inhibitory postsynaptic potentials carry synchronized frequency information in active cortical networks. Neuron.

[b16] Major G, Tank D (2004). Persistent neural activity: prevalence and mechanisms. Curr. Opin. Neurobiol.

[b17] Mann EO, Kohl MM, Paulsen O (2009). Distinct roles of GABA_A_ and GABA_B_ receptors in balancing and terminating persistent cortical activity. J. Neurosci.

[b18] Petersen CC (2003). The barrel cortex - integrating molecular, cellular and systems physiology. Pflugers Arch.

[b19] Plotkin JL, Day M, Surmeier DJ (2011). Synaptically driven state transitions in distal dendrites of striatal spiny neurons. Nat. Neurosci.

[b20] Rosenkranz JA, Johnston D (2006). Dopaminergic regulation of neuronal excitability through modulation of *I*_h_ in layer V entorhinal cortex. J. Neurosci.

[b21] Sanchez-Vives MV, McCormick DA (2000). Cellular and network mechanisms of rhythmic recurrent activity in neocortex. Nat. Neurosci.

[b22] Schultz W (2007). Multiple dopamine functions at different time courses. Annu. Rev. Neurosci.

[b23] Seamans JK, Yang CR (2004). The principal features and mechanisms of dopamine modulation in the prefrontal cortex. Prog. Neurobiol.

[b24] Seamans JK, Durstewitz D, Christie BR, Stevens CF, Sejnowski TJ (2001). Dopamine D1/D5 receptor modulation of excitatory synaptic inputs to layer V prefrontal cortex neurons. Proc. Natl. Acad. Sci. USA.

[b25] Shu Y, Hasenstaub A, McCormick DA (2003). Turning on and off recurrent balanced cortical activity. Nature.

[b26] Steriade M, Nunez A, Amzica F (1993). A novel slow (< 1 Hz) oscillation of neocortical neurons in vivo: depolarizing and hyperpolarizing components. J. Neurosci.

[b27] Towers SK, Hestrin S (2008). D_1_-like dopamine receptor activation modulates GABAergic inhibition but not electrical coupling between neocortical fast-spiking interneurons. J. Neurosci.

[b28] Weiss T, Veh RW, Heinemann U (2003). Dopamine depresses cholinergic oscillatory network activity in rat hippocampus. Eur. J. Neurosci.

